# Investigation of Three Newly Identified Equine Parvoviruses in Blood and Nasal Fluid Samples of Clinically Healthy Horses and Horses with Acute Onset of Respiratory Disease

**DOI:** 10.3390/ani11103006

**Published:** 2021-10-19

**Authors:** Nicola Pusterla, Kaitlyn James, Samantha Barnum, Eric Delwart

**Affiliations:** 1 Department of Medicine and Epidemiology, School of Veterinary Medicine, University of California, Davis, CA 95616, USA; smmapes@ucdavis.edu; 2 Department of Obstetrics, Gynecology and Reproductive Biology, Massachusetts General Hospital, Boston, MA 02114, USA; kaitlynej@gmail.com; 3 Vitalant Research Institute, San Francisco, CA 94118, USA; Eric.Delwart@ucsf.edu; 4 Department of Laboratory Medicine, University of California, San Francisco, CA 94118, USA

**Keywords:** equine parvoviruses, equine parvovirus hepatitis, equine parvovirus CSF, equine copivirus, nasal fluid, blood, qPCR, sick equids, healthy horses

## Abstract

**Simple Summary:**

The objective of the present study was to determine the molecular frequency of three recently identified parvoviruses (equine parvovirus hepatitis, equine parvovirus CSF and equine copivirus) in blood and respiratory secretions of 667 equids with acute onset of fever and respiratory signs and 87 clinically healthy horses. One hundred and seventeen sick horses tested qPCR-positive for at least one of the three parvoviruses. Ten clinically healthy horses tested qPCR-positive for one of the equine parvoviruses. The frequency of detection of the three equine parvoviruses was similar between sick and clinically healthy horses, suggesting that these newly characterized viruses do not appear to contribute to the clinical picture of equids with respiratory disease. In order to prove the clinical relevance of any of these newly identified equine parvoviruses, experimental challenge studies using pure, clonal inocula will be required.

**Abstract:**

Three newly identified equine parvoviruses (equine parvovirus hepatitis (EqPV-H), equine parvovirus CSF (EqPV-CSF) and equine copivirus (Eqcopivirus)) have recently been discovered in horses with respiratory signs. However, the clinical impact of these three equine parvoviruses has yet to be determined. Nasal fluid samples and blood from 667 equids with acute onset of fever and respiratory signs submitted to a diagnostic laboratory were analyzed for the presence of common equine respiratory pathogens (equine influenza virus, equine herpesvirus-1/-4, equine rhinitis A and B virus, *S. equi* subspecies *equi*) as well as EqPV-H, EqPV-CSF and Eqcopivirus by qPCR. An additional 87 clinically healthy horses served as controls. One hundred and seventeen sick horses tested qPCR-positive for at least one of the three parvoviruses. Co-infections with common respiratory pathogens and parvoviruses were seen in 39 sick equids. All 87 clinically healthy horses tested qPCR-negative for all tested common respiratory pathogens and 10 healthy horses tested qPCR-positive for one of the equine parvoviruses. When the frequency of detection for EqPV-H, EqPV-CSF and Eqcopivirus of equids with respiratory signs was compared to that of clinically healthy horses, the difference was not statistically significant (*p* > 0.05), suggesting that the three recently identified equine parvoviruses do not contribute to the clinical picture of equids with respiratory disease.

## 1. Introduction

Equine infectious respiratory diseases represent one of the most common clinical entities reported by practicing veterinarians nationwide [1], with equine influenza virus (EIV), equine herpesvirus-1 (EHV-1), EHV-4 and equine rhinitis A (ERAV) and B (ERBV) viruses being considered the leading respiratory viruses [2,3,4,5]. The list of newly identified respiratory viruses in humans and various animal species has in the past decade expanded with the introduction of metagenomics [6,7]. This approach uses viral particle enrichment, random nucleic acid amplification and deep sequencing followed by bioinformatics analysis for the presence of viral sequences [8]. Two studies have recently reported three new equine parvoviruses, named equine parvovirus hepatitis (EqPV-H), equine parvovirus CSF (EqPV-CSF) and equine copivirus (Eqcopivirus) [9,10]. These equine parvoviruses were identified in blood and nasal secretions of apparently healthy horses and horses with acute onset of respiratory signs. However, these studies were unable to demonstrate causality for these newly identified equine parvoviruses. It is, therefore, the aim of this study to determine the frequency of genome detection of three newly identified parvoviruses (EqPV-H, EqPV-CSF and Eqcopivirus) in blood and nasal fluid samples of horses with acute onset of fever and respiratory signs, as well as clinically healthy control horses, and to determine potential demographic and clinical prevalence factors associated with these parvoviruses.

## 2. Materials and Methods

### 2.1. Study Population and Sampling

Blood and nasal fluid samples from 667 horses, mules and donkeys with acute onset of fever and respiratory signs were enrolled in the study. The samples were submitted to the Real-Time PCR Research and Diagnostics Core Facility, School of Veterinary Medicine, University of California at Davis from 1 January 2020 to 31 December 2020. Demographic and clinical information was gathered from the submission forms. Additional blood and nasal fluid samples from 87 clinically healthy horses were collected during the same period to determine the rate of equine parvoviruses in this population. The clinically healthy horses presented to the William R. Pritchard Veterinary Medical Teaching Hospital, School of Veterinary Medicine, University of California at Davis for routine health care (vaccination, health care certificate, oral examination) or elective procedures. Clients were asked to fill out a consent form allowing the collection of biological samples.

### 2.2. DNA Purification and Quantitative PCR Analyses

Nucleic acid extraction from whole blood and nasal fluid samples was performed using an automated nucleic acid extraction system (QIAcubeHT, Germantown, MD, USA) according to the manufacturer’s recommendations. For blood samples, 100 µL of whole blood was processed for nucleic acid purification. The collected nasal swabs were placed into a conical tube containing 1000 µL of phosphate-buffered saline (PBS), vortexed and quickly centrifuged in order to release the nasal fluid from the swab. A total of 200 µL of nasal fluid/PBS solution was processed for nucleic acid purification.

Nasal fluid samples from sick and clinically healthy horses were tested for the presence of common respiratory pathogens, including EIV, EHV-1, EHV-4, equine rhinitis A and B virus (ERVs) and *S. equi*, as previously reported [2,11]. Blood from the same horses was tested for EHV-1 [2]. Further, nasal fluid samples and blood of all study horses were tested for EqPV-H, EqPV-CSF and Eqcopivirus using established and validated qPCR assays. Published sequences from GenBank (www.ncbi.nlm.nih.gov/genbank, accessed on 16 September 2019) of all three equine parvoviruses were subjected to BLAST analysis, and the aligned sequences were used to design primers and probes (Table 1). The samples were amplified in a combined thermocycler/fluorometer (7900 HT Fast, Applied Biosystems, Foster City, CA, USA) with the standard thermal cycling protocol: 2 min at 50 °C, 10 min at 95 °C and 40 cycles of 15 s at 95 °C and 60 s at 60 °C. The PCR reactions for each of the three parvovirus assays was composed of a commercially available mastermix (Universal TaqMan Mastermix with AmpErase UNG, Applied Biosystems, Foster City, CA, USA), containing 10 mM Tris (pH 8.3), 50 mM KCl, 5 mM MgCl_2_, 300 μM each of dATP, dCTP and dGTP, 600 μM dUTP, 0.625 U of AmpliTaq Gold per reaction, 0.25 U AmpErase UNG per reaction, 400 nM of each primer and 80 nM of the respective TaqMan probe and 1 μL of DNA sample for a total volume of 12 μL. For each of the three parvoviruses, standard curves were generated using synthetic long oligonucleotides containing the target sequence for EqPV-H, EqPV-CSF and Eqcopivirus, and the amplification efficiency was calculated from the slope using the formula E = 10^[−1/slope]^. The amplification efficiency was 99%, 100% and 95% for the capsid protein gene of EqPV-H, the capsid VP1 gene of EqPV-CSF and the NS1 gene of Eqcopivirus, respectively, indicating a very high analytical sensitivity. The detection limit for the three parvovirus assays was 13 genome equivalents when the DNA was purified from nasal fluid samples and whole blood. To determine the quality and efficiency of nucleic acid extraction, all samples were analyzed for the presence of the housekeeping gene equine glyceraldehyde-3-phosphate dehydrogenase (eGAPDH), as previously described [2].

### 2.3. Statistical Analyses

Descriptive analyses (mean, standard deviation and median) were performed to evaluate the demographic and clinical information from the submission forms. Categorical analyses were performed using a Pearson’s chi-square test to determine the association between observations (age, breed, sex, clinical signs (rectal temperature, nasal discharge, coughing) and infections. Each parvovirus infectious disease group was compared to non-parvovirus infected sick horses. To avoid interpretation bias when multiple pathogens were involved, only horses with a single pathogen (parvoviruses and non-parvoviruses) were evaluated. All statistical analyses were performed using commercial software (Stata Statistical Software, Version 14, College Station, TX, USA) and statistical significance was set at *p* < 0.05.

## 3. Results

Demographic and clinical information (age, breed and sex) was available for 453/667 sick equids (68%). The age of the sick equids ranged from 1 month to 34 years, with a median of 9 years. Sixty-one percent of the equids were males (stallions and geldings), and 39% were females. A variety of breeds were represented, including Quarter Horse (37%), Warmblood (14%), Thoroughbred (10%), pony breed (6%), Arabian (5%), Paint Horse (4%) and other breeds (22%). There were 12 donkeys and 3 mules (2%) reported. Clinical signs included fever (range 38.6 to 41.4 °C, median 39.4 °C) in 97%, nasal discharge in 74% and coughing in 46% of equids with reported clinical signs. The population of clinically healthy horses was composed of 50 males (57%) and 37 females (43%) with ages from 3 months to 32 years (median 7.5 years).

The frequency of detection for common respiratory pathogens in sick equids was as follows: 81 EIV (12.1%), 61 *S. equi* (9.1%), 50 EHV-4 (7.5%), 36 ERVs (5.4%) and 13 EHV-1 (1.9%, Table 2). Four equids EHV-1 qPCR-positive in nasal fluid samples also tested positive for EHV-1 in blood. Overall, 48 equids tested qPCR-positive for EqPV-H (2 nasal fluid samples only, 40 blood only, 6 both nasal fluid samples and blood). For EqPV-CSF, 35 equids tested qPCR-positive (4 nasal fluid samples only, 27 blood only, 4 both nasal fluid samples and blood). Fifty-nine equids tested qPCR-positive for Eqcopivirus (10 nasal fluid samples only, 24 blood only, 25 both nasal fluid samples and blood). Amongst the 117 equids with equine parvovirus infection, 95 had a single infection (46 Eqcopivirus, 29 EqPV-H and 20 EqPV-CSF), 20 had dual infections (10 EqPV-H and EqPV-CSF, 7 Eqcopivirus and EqPV-H and 3 Eqcopivirus and EqPV-CSF) and 2 had triple infections (Eqcopivirus, EqPV-H and EqPV-CSF). Co-infections with common respiratory pathogens and parvoviruses were seen in 39 equids (15 *S. equi*, 11 EIV, 11 ERVs, 8 EHV-4 and 1 EHV-1). All 87 clinically healthy horses tested qPCR negative for EIV, EHV-1, EHV-4, ERVs and *S. equi*. Ten clinically healthy horses tested qPCR positive for one of the equine parvoviruses (5 EqPV-H, 4 Eqcopivirus and 1 EqPV-CSF; Table 2).

When demographic prevalence factors were determined for each of the infectious groups (common respiratory pathogen and parvoviruses), the median age for EHV-4 and ERVs was significantly lower compared to most of the other groups (*p* < 0.05; Table 3). The EqPV-H and EqPV-CSF groups had the highest median age population with 10 and 15 years of age, respectively. There were more male than female equids in the various groups, although the differences were not statistically significant (*p* > 0.05). While the rectal temperature for the sick horses showed a wide range from 36.9 to 41.4 °C, there were no differences in median rectal temperatures amongst the various infectious groups. The reported frequency of nasal discharge for the various infectious groups ranged from 61.1% to 90.2%, with the lowest frequencies found in the three parvovirus groups. Equids infected with EIV had a significantly higher frequency of reported nasal discharge when compared to equids from the EHV-4, EqPV-H, EqPV-CSF, Eqcopivirus and the negative infection group (*p* < 0.01). The frequency of coughing ranged from 22.2% to 90.2%, with the highest frequency found in the EIV infection group and the lowest in the EHV-1 infection group. The EIV infection group had a significantly higher frequency of reported coughing compared to all the other infection groups, with the exception of the ERVs group (*p* < 0.01).

When the frequency of detection of EqPV-H, EqPV-CSF and Eqcopivirus, in plasma and nasal fluid samples of equids with respiratory signs was compared to that in plasma and nasal fluid samples of healthy horses, the difference was not statistically significant (*p* > 0.05).

## 4. Discussion

Although the three newly identified equine parvoviruses have been characterized from biological samples of both clinically diseased and clinically healthy horses, their clinical relevance has remained elusive. The best-investigated equine parvovirus is EqPV-H, which has been linked to clinical and subclinical hepatitis [12,13,14,15,16]. EqPV-H has been shown to infect and replicate in hepatocytes, and viral infection is associated with liver pathology during hepatitis [11,15]. Further, EqPV-H has been detected in oral and nasal secretions and feces of experimentally infected horses, suggesting that these biological samples may be involved in horizontal transmission [15]. While EqPV-H has been experimentally transmitted via the oral route in one single horse, the intranasal inoculation of EqPV-H containing serum in two horses did not lead to viremia [15]. However, other transmission routes, such as the oral, nasal and vector-borne route, remain possible, especially around peak viremia [15,16]. Based on a recent study, vertical transmission does not appear to be a major contributor to the epidemiology of EqPV-H [14]. Reports on EqPV-CSF and Eqcopivirus have been sparse since the first description of these equine parvoviruses [9,10]. EqPV-CSF was initially found in the cerebrospinal fluid of a horse with neurological signs and has since then been reported in serum and nasal secretions of both clinically healthy horses and horses with fever and respiratory signs [9,10,17]. To the authors’ knowledge, only one single report has documented the genomic presence of Eqcopivirus in blood and/or nasal secretions of healthy horses and horses with respiratory signs [10]. The detection of any of the three newly identified equine parvoviruses in nasal fluid samples is relevant, as viral shedding can contribute to environmental contamination with direct or indirect transmission to susceptible equids.

One hundred and seventeen horses (17%) with acute onset of fever and respiratory signs tested qPCR-positive for at least one of the three parvoviruses. Blood was the predominant sample positive for EqPV-H and EqPV-CSF, while blood and nasal fluid samples tested qPCR-positive for Eqcopivirus with similar frequencies. It appears that the three newly identified equine parvoviruses may have a species affinity, as all 15 donkeys and mules tested qPCR-negative for the three parvoviruses. While a large population of non-horse equids will need to be screened to strengthen the parvovirus species-specificity, the present study results are in agreement with a previous study, which was unable to detect EqPV-H in 13 donkeys from Austria [18].

One of the characteristics of a respiratory virus is its tropism to epithelial cells and the transient shedding in respiratory secretions during clinical or subclinical disease. Neither EqPV-H nor EqPV-CSF showed frequent detection by qPCR in nasal secretions, which agrees with a previous study [10]. The low detection rate of these two parvoviruses in nasal fluid samples reduces the likelihood that these two viruses are associated with clinical respiratory disease. Further, the frequency of detection of EqPV-H and EqPV-CSF in sick equids was similar to the one detected in clinically healthy horses. While there was no difference in the detection of Eqcopivirus between sick and clinically healthy equids, this virus had the highest detection rate in nasal fluid samples compared to the two other parvoviruses. Even if Eqcopivirus is not associated with clinical respiratory disease, the high detection rate observed in the present study population may relate to the spread of this virus via nasal secretions and possible transmission via droplets.

The study results showed that horses with active parvovirus infection were significantly older compared to horses infected with common respiratory pathogens. These results are in agreement with two recent studies, which determined that age was a risk factor influencing the rate of EqPV-H infections [18,19]. The study by Badenhorst and colleagues [18] determined that with every increase of 1 year in age, the risk of active EqPV-H infection was 1.1 times higher. Other demographic and clinical risk factors associated with any of the three equine parvoviruses were not any different than for horses presenting with fever, nasal discharge and coughing and having no common respiratory pathogens detected by qPCR. The similarity of prevalence factors between the parvovirus qPCR-positive equids and sick equids without detectable respiratory pathogens in nasal secretions further reinforces the lack of causality between these three equine parvoviruses and respiratory disease in the present study population.

The frequency of detection of the three parvoviruses in plasma and nasal fluid samples was comparable between horses with respiratory disease and clinically healthy horses. Further, the DNA prevalence for EqPV-H, EqPV-CSF and Eqcopivirus detected in the study population of sick and clinically healthy equids was similar to previously published studies. Surveillance studies in healthy horses from the USA, China, Austria and Germany have reported DNA prevalence for EqPV-H ranging between 7.1% and 17.0% [10,12,18,19,20,21]. The frequency of detection of EqPV-CSF in sick and clinically healthy horses was similar to the 4.9% detection rate reported in 41 healthy horses from the USA [10] and lower than the 23.1% reported from healthy Thoroughbred horses undergoing a custom quarantine in North Xinjiang province, China [17]. The prevalence of Eqcopivirus in plasma and or nasal fluid samples from clinically healthy equids in the present study was lower than the previously reported prevalence of 17% determined in plasma samples from apparently healthy horses [10]. Differences in horse populations used for the various studies are likely the reason for the observed differences in equine parvovirus detection rates.

Limitations of the study relate to the inability to re-sample the study animals. Longitudinal monitoring of affected equids would allow determining viral outcome following the convalescent period. One additional limitation was that the clinically healthy control population included in this study was not matched for age, time and location.

## 5. Conclusions

In conclusion, while EqPV-H, EqPV-CSF and Eqcopivirus can be found predominantly in the blood of equids with acute onset of fever and respiratory signs, it does not appear that these three newly identified parvoviruses contribute to the clinical picture of equids with respiratory disease. Since the initial characterization of EqPV-H, EqPV-CSF and Eqcopivirus, two additional equine parvoviruses and a previously unknown picornavirus have been described in the tissues of horses with interstitial pneumonia [22]. In order to prove the clinical relevance of any of these new equine parvoviruses, experimental challenge studies using pure, clonal inocula will be required.

## Figures and Tables

**Table 1:** Oligonucleotide sequences of primers and probes used to detect three newly identified equine parvoviruses by qPCR.

Equine Parvovirus	Target Gene (GenBank)	Oligonucleotides
EqPV-H	Capsid protein	EqPV-H-forward primer: AGAATGCAGATGCTTTCCGAC
	(MH500792)	EqPV-H-reverse primer: AAAGCAGATCCCGAATCCG
		EqPV-H-probe: FAM-GAAGATTCATGAGCTAGTC-MGB
EqPV-CSF	Capsid VP1	EqPV-CSF-forward primer: AAGGCTTTGGACAAACGGG
	(KR902500)	EqPV-CSF-reverse primer: TTGTTAGCACATGCGTTCCC
		EqPV-CSF-probe: FAM-AAGGGATATGGAAGGGA-MGB
Eqcopivirus	NS1	EqCopi-forward primer: TCGCCCAGATCGTTGAGAAC
	(MN181468)	EqCopi-reverse primer: AGCTGCTGTCTCCTGTTGTCC
		EqCopi-probe: FAM-ACCCAATCACCGAAGC-MGB

**Table 2:** Frequency of detection of common respiratory pathogens and newly identified equine parvoviruses in sick and clinically healthy equids.

Pathogen	Sick Equids (667)		Clinically Healthy Horses (87)	
	Nasal Fluid	Blood	Nasal Fluid	Blood
EIV	81 (12.1%)	Not tested	0	Not tested
*S. equi*	61 (9.1%)	Not tested	0	Not tested
EHV-4	50 (7.5%)	Not tested	0	Not tested
ERVs	36 (5.4%)	Not tested	0	Not tested
EHV-1	13 (1.9%)	4 (0.6%)	0	0
EqPV-H	8 (1.2%)	46 (6.9%)	1 (1.1%)	5 (5.7%)
EqPV-CSF	8 (1.2%)	32 (4.8%)	0	1 (1.1%)
Eqcopivirus	35 (5.2%)	49 (7.3%)	2 (2.3%)	4 (4.6%)

**Table 3:** Demographic and clinical data from 667 equids with acute onset of fever and/or respiratory signs. The data is presented for each common respiratory pathogen and the three newly identified equine parvoviruses. Only animals with a single detected pathogen are reported. Demographic and clinical information was available for approximately 68% of the sick equids.

Pathogen	Demographic		Clinical Signs		
	Age Range in Years (Median)	Sex Distribution (Male/Female)	Rectal Temperature Range in °C (Median)	Nasal Discharge (%)	Coughing (%)
EHV-1 (9)	1–12 (5)	5/4	38.8–40.9 (39.8)	6/9 (66.7)	2/9 (22.2)
EHV-4 (35)	1–23 (4)	20/15	38.6–40.9 (39.6)	24/35 (68.6)	12/35 (34.3)
EIV (61)	1–22 (8)	28/33	38.6–41.1 (39.3)	55/61 (90.2)	55/61 (90.2)
ERVs (17)	1–25 (3)	9/8	38.6–40.8 (39.4)	13/17 (76.5)	10/17 (58.8)
*S. equi* (37)	2–22 (7)	23/14	38.7–41.0 (39.4)	31/37 (83.8)	16/37 (43.2)
Eqcopivirus (31)	1–30 (8.5)	16/15	38.1–41.4 (39.4)	20/31 (64.5)	14/31 (45.2)
EqPV-H (18)	1–30 (10)	11/7	38.0–40.6 (39.4)	11/18 (61.1)	8/18 (44.4)
EqPV-CSF (13)	1–26 (15)	8/5	36.9–40.0 (39.3)	8/13 (61.5)	7/13 (53.8)
Negative (376)	1–34 (9)	206/170	37.2–41.2 (39.4)	262/376 (69.7)	137/376 (36.4)

## Data Availability

Data available on request due to privacy restrictions.
